# Factors and Barriers on Cardiopulmonary Resuscitation and Automated External Defibrillator Willingness to Use among the Community: A 2016–2021 Systematic Review and Data Synthesis

**DOI:** 10.5334/gh.1255

**Published:** 2023-08-25

**Authors:** Amsyar Daud, Azmawati Mohammed Nawi, Azimatun Noor Aizuddin, Mohammad Fadhly Yahya

**Affiliations:** 1 Department of Public Health Medicine, Faculty of Medicine, National University of Malaysia, Kuala Lumpur 56000, Malaysia; 2 Emergency and Trauma Department, Malacca General Hospital, Jalan Mufti Haji Khalil, Malacca 75450, Malaysia

**Keywords:** cardiopulmonary resuscitation, automated external defibrillator, factor, barrier, community willingness

## Abstract

**Background:**

Bystander cardiopulmonary resuscitation (CPR) and using an automated external defibrillator (AED) can improve out-of-hospital cardiac arrest survival. However, bystander CPR and AED rates remained consistently low. The goal of this systematic review was to assess factors influencing community willingness to perform CPR and use an AED for out-of-hospital cardiac arrest survival (OHCA) victims, as well as its barriers.

**Methods:**

The review processes (PROSPERO: CRD42021257851) were conducted following the Preferred Reporting Items for Systematic Reviews and Meta-Analyses (PRISMA) review protocol; formulation of review questions; systematic search strategy based on identification, screening, and eligibility using established databases including Scopus, Web of Science, and Medline Complete via EBSCOhost; quality appraisal; and data extraction and analysis. There is identification of full-text journal articles that were published between 2016 and 2021 and written in English.

**Results:**

Of the final 13 articles, there are six identified factors associated with willingness to perform CPR and use an AED, including socio-demographics, training, attitudes, perceived norms, self-efficacy, and legal obligation. Younger age, men, higher level of education, employed, married, having trained in CPR and AED in the previous 5 years, having received CPR education on four or more occasions, having a positive attitude and perception toward CPR and AED, having confidence to perform CPR and to apply an AED, and legal liability protection under emergency medical service law were reasons why one would be more likely to indicate a willingness to perform CPR and use an AED. The most reported barriers were fear of litigation and injuring a victim.

**Conclusions:**

There is a need to empower all the contributing factors and reduce the barrier by emphasizing the importance of CPR and AEDs. The role played by all stakeholders should be strengthened to ensure the success of intervention programs, and indirectly, that can reduce morbidity and mortality among the community from OHCA.

## 1. Introduction

Out-of-hospital cardiac arrest (OHCA) is one of the leading causes of death in developed countries, where more than 135 million deaths are recorded annually. OHCA is considered a major burden on the population [1,2,3]. Each year, more than 275,000 cases involving OHCA are recorded in Europe, more than 600,000 cases involving OHCA occur in the United States, about 550,000 occur cases in China. Of these cases, less than 15% survive [4,5,6,7].

Community involvement through early recognition of cardiac arrest, activating the EMS system, bystander cardiopulmonary resuscitation (CPR), and using an automated external defibrillator (AED) can improve OHCA survival [8,9]. CPR is a life-saving medical intervention that improves the chance of survival following cardiac arrest [10]. Besides, an AED is a portable, lifesaving device that can be used by the general public or trained professionals, and it uses voice prompts to guide the CPR provider to treat sudden cardiac arrest [11,12]. AED is effective in saving lives when used quickly following collapse, safe when used by laypeople with minimal or no training, and accurate. It will deliver a shock only when ventricular fibrillation (VF) or rapid ventricular tachycardia (VT) is present from sudden cardiac arrest victims [11,13].

Early CPR can increase patient survival two to four times, and with the use of AED, it can increase survival by 50–70% with good neurological outcomes. CPR is associated with higher hospital discharge rates [11,14,15,16,17]. Therefore, the improvements in bystander CPR and AED are associated with increased survival [17].

The issue of community willingness to respond to OHCA events involving CPR and the use of an AED has piqued the interest of researchers worldwide. Understanding the factors and barriers is critical to improving OHCA survival. Despite the fact that there is a large body of literature on CPR and AED willingness at the moment, there has been little effort put into systematically reviewing these studies, identifying patterns, and developing potential themes on the subject. The goal of this systematic review is to assess the factors influencing community willingness to perform CPR and use an AED for OHCA victims, as well as its barriers. The authors were guided through the review by the main research question: ‘What are the factors and barriers on the community’s willingness to perform CPR and use an AED?’

## 2. Methods

### 2.1 Study registration

The review was conducted following the PRISMA [18]. This systematic review has been registered on PROSPERO (Registration number: CRD42021257851, 28 June 2021).

### 2.2 Review question

Our research question in this study was formulated using the Population, Prognostic Factors and Outcome (PFO) strategy [19]. Based on these concepts, three main aspects were included in the review, as shown in Table 1.

**Table 1:** PFO strategy in developing research question.


**Population (P)**	Community adults, age 19 years and above

**Prognostic Factors (F)**	The factors or barriers that could influence the outcome (community willingness to perform CPR and using an AED)

**Outcome (O)**	Community willingness to perform CPR and using an AED


### 2.3 Search strategy and information source

A systematic literature search strategy was employed in this review to retrieve complete and comprehensive articles related to the research topic. A detailed protocol was used for the systematic review, whereby all the relevant studies obtained from the literature search were extracted and examined. This procedure step was run systematically in seven steps as follows: develop a research question; select suitable keywords in relation to the topics; define the databases that are relevant for the search process; determine the limitations of the search, the languages used, and the nature of the documents; develop a review strategy; screen and examine the desired literature; and analyze the selected articles.

To select the number of articles relevant to the research topic, the identification of keywords was conducted, which aimed to provide more options and selection of databases to search for more related studies. All similar terms to the keywords and phrases were identified using an online thesaurus dictionary, multiple synonyms and terminology variations, medical subject heading (MeSH) terms for each concept, and past research articles. These terms will be executed using advanced searching techniques such as Boolean operators ‘and/or,’ phrase searching, wild cards, appropriate truncation, and phrase symbols to form initial search strategies, followed by combining these searching techniques into a full searching string. The main databases comprising Scopus, Web of Sciences (WOS), and Medline Complete via Ebscohost were searched for articles relevant to the topic. The detailed, full search string strategy used in Scopus, WOS, and Medline Complete via Ebscohost is shown in Appendix 1.

**Figure 1:**
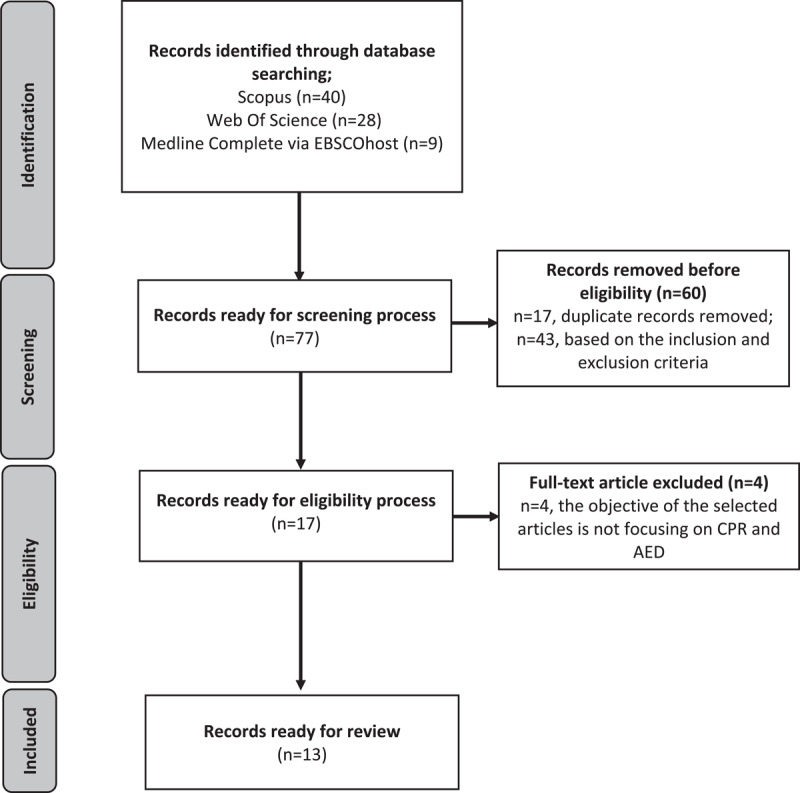
Flow diagram of the searching process.

The titles and abstracts of each article were examined for relevance and screened based on specific criteria by the authors. The timeline was set to 2016–2021 because the number of published studies was sufficient to perform a representative review. The inclusion criteria for article selection were: (1) published between 2016 and 2021, (2) a full journal article, and (3) written in English. Articles of systematic review, conference proceedings, book chapters, and reports were excluded. There were 77 articles identified through database searching (Figure 1). There were 17 full-text articles successfully retrieved for eligibility. The authors reviewed all full text articles and recorded the reason for the article’s exclusion. A total of 4 articles were excluded due to the objective of the selected articles not focusing on CPR and AED. The remaining articles (n = 13) went forward with the quality appraisal process.

### 2.4 Quality appraisal

The authors evaluated the articles using a Mixed-Method Appraisal Tool (MMAT) [20]. These tools were selected based on their capacities to appraise quantitative, qualitative, and mixed-method study designs. The authors then evaluated the methodological and analytical rigor of each article with the assistance of two co-authors. Each article was thoroughly read, with a particular emphasis on the methodology section and the analysis performed. The authors scrutinized the articles guided by MMAT (Appendix 2).

Each article was appraised on five criteria, with three potential responses: ‘yes,’ ‘no,’ and ‘don’t know/can’t tell.’ The review included articles that met at least three of the criteria. All assessment decisions were made unanimously, and any disagreements were quickly resolved through discussion among the authors to avoid cases of dispute. All authors agreed that all selected articles met the minimum quality requirement for methodology and analysis as a result of this process. In total, 11 articles fulfilled all the criteria, one article fulfilled at least four criteria, and one article managed to fulfil at least three criteria. The results of the quality assessment are shown in Table 2.

**Table 2:** Results of the quality assessment.


STUDY	RESEARCH DESIGN	QA1	QA2	QA3	QA4	QA5	NUMBER OF CRITERIA FULFILLED	INCLUSION IN THE REVIEW

Bylow et al. [21]	QN (R)	Y	Y	Y	Y	Y	5/5	✓

Cheskes et al. [22]	QN (DC)	Y	Y	Y	Y	Y	5/5	✓

Fan et al. [23]	QN (DC)	Y	Y	Y	Y	Y	5/5	✓

Hawkes et al. [24]	QN (DC)	Y	Y	Y	Y	Y	5/5	✓

Krammel et al. [25]	QN (NR)	Y	N	Y	C	Y	3/5	✓

Lee et al. [26]	QN (NR)	Y	Y	Y	Y	Y	5/5	✓

Liaw et al. [27]	QN (NR)	Y	Y	Y	N	Y	4/5	✓

Pei-Chuan Huang et al. [28]	QN (DC)	Y	Y	Y	Y	Y	5/5	✓

Pei-Chuan Huang et al. [29]	QN (DC)	Y	Y	Y	Y	Y	5/5	✓

Post et al. [30]	QN (NR)	Y	Y	Y	Y	Y	5/5	✓

Qian et al. [31]	QN (DC)	Y	Y	Y	Y	Y	5/5	✓

Shams et al. [32]	QN (DC)	Y	Y	Y	Y	Y	5/5	✓

Son et al. [33]	QN (DC)	Y	Y	Y	Y	Y	5/5	✓


Notes: QL = Qualitative; QN (R) = Quantitative randomized controlled trials; QN (NR) = Quantitative non-randomized; QN (DC) = Quantitative descriptive; MX = Mixed-Method; Y = Yes; N = No; C = Can’t tell.

## 3. Results

### 3.1 Background of the eligible studies

From 13 articles, 2 papers focused their studies on Taiwan [28,29], 2 on South Korea [26,33], and one on Sweden [21], Canada [22], Hong Kong [23], United Kingdom [24], Austria [25], Malaysia [27], United States [30], China [31], and Lebanon [32]. All 13 studies focused on quantitative analyses. Regarding the year of publication, three articles were published in 2016 [22,23,32], one article was published in 2017 [33], one article was published in 2018 [25], four articles were published in 2019 [21,24,28,30], one article was published in 2020 [27], and three articles was published in 2021 [26,29,31].

Based on study settings, ten studies were conducted among a community [21,22,23,24,25,26,28,29,31,33], one were based in a school [30], and two were in a university [27,31]. For the targeted population, all studies were conducted among adults. The included studies indicated which type of collection method was used: self-administered questionnaire (n = 4, 31%), face-to-face interview survey (n = 3, 23%), online survey (n = 3, 23%), and telephone survey (n = 3, 23%).

Table 3 shows the summary of the findings in the included studies by the different countries and populations, different types of interventional tools, and the identified factors and barriers against performing CPR and using an AED.

**Table 3:** A summary of the studies included in this study.


FIRST AUTHOR (YEAR)COUNTRY, PERIOD OF STUDY	STUDY COVERAGE	SAMPLE SIZE AND POPULATION	INTERVENTION	COMPARISON	FACTORS ASSOCIATED WITH WILLINGNESS	BARRIERS

Bylow (2019) Sweden, 2014–2016	BLS (CPR and AED)	1,231 Public in the community	Basic life support training intervention	To compare the effectiveness of two BLS training intervention, self-learning training or traditional instructor-led training	Training	Not applicable

Cheskes (2016) Canada, 1 week	CPR	428 Adults living in Canada	Not applicable	Chest-compression-only CPR (CCO-CPR) compared to traditional CPR with mouth-to-mouth (MTM) ventilations	Gender, type of CPR training	Lack of knowledge, fear of litigation, lack of skill confidence

Fan (2016) Hong Kong, 2 November–15 December 2015 (excluding Saturdays and public holidays)	AED	401 All pedestrians in the vicinity of a mass transit railway (MTR) station in six locations across different districts of Hong Kong	Not applicable	Comparison of first aiders with non–first aiders	Training	Not applicable

Hawkes (2019) United Kingdom, May 2017	CPR and PAD	2,084 UK adults	Not applicable	CCO-CPR, CPR, and Public Access Defibrillator (PAD) training	Age, gender, occupation, marital status, training, length of time since last training, confidence in performing CPR	Not applicable

Krammel (2018) Austria, August–September 2014	BLS (CPR and AED)	501 Residents of Vienna (Austria)	Not applicable	Comparison of gender and age-specific aspects of awareness and knowledge	Age, gender	Not applicable

Lee (2021) South Korea, February 2012, December 2016, and December 2018	AED	3,069 Daegu Metropolitan City, South Korea	Not applicable	973 in the 2012 survey (first group), 1,095 in the 2016 survey (second group), and 1,001 in the 2018 survey (third group)	Gender, length of time since last training, legal obligation, awareness of how to use an AED	Lack of knowledge, fear of litigation, injuring victim due to using AED, not interested in AED

Liaw (2020) Malaysia, February–November 2018	CPR and AED	184 Employees from UNIMAS, Malaysia	Training program on how to use the AED and perform CPR	To determine the effectiveness of the training program, to improve the perception of using AED and performing CPR (‘pre-test’ vs. ‘post-test’)	Training, perception, confidence behavior	Fear of disease transmission, injuring victim due to performing CPR, injuring own self due to performing CPR, injuring victim due to using AED, injuring own self due to using AED

Pei-Chuan Huang (2019) Taiwan, April–June 2013	CPR	1,073 Public in Taiwan	Not applicable	Not applicable	Gender, training, legal obligation	Fear of legal responsibilities, injuring victim due to performing CPR

Pei-Chuan Huang (2021) Taiwan, April–June 2013	AED	1,073 Public in Taiwan	Not applicable	Not applicable	Gender, training, attitude	Fear of legal responsibilities, lack of skill confidence, injuring victim due to using AED

Post (2019) United States	CPR and AED	769 Coaches of high school teams and club teams with high school-aged athletes in 3 sports (basketball, soccer, and volleyball)	High school coaches were required to undergo annual CPR/AED training	Emergency preparedness and training existed between coaches of high school teams and coaches of high school-aged club teams	Training	Not applicable

Qian (2021) People’s Republic of China, October–December 2020	BLS (CPR and AED)	2,812 Urban residents in Nantong City	None	Residents with first aid experience and residents without first aid experience	Age, education level, occupation, training	Fear of legal responsibilities, fear of disease transmission, injuring victim due to performing CPR, performing MTM artificial respiration, concern that peoples around them will complain, unwillingness to touch strangers

Shams (2016) Lebanon, 1 March–30 June 2015	CPR and AED	948 University students in Lebanon	Not applicable	Not applicable	Social grade, training, confidence in performing CPR	Lack of knowledge, fear of disease transmission, injuring victim due to performing CPR, poor hygiene of victim, presence of vomit or blood

Son (2017) South Korea, 3–14 February 2012	CPR and AED	1,000 Adults aged 19 years or above living in Daegu metropolitan city.	Not applicable	Type of CPR education, total no. of CPR education sessions attended, period from the last CPR education session, AED training included in CPR education	Age, gender, education level, type of CPR training, length of time since last training, number of times trained, confidence in performing CPR, legal obligation	Not applicable


### 3.2 Factors associated with community willingness

The review found multiple factors have been frequently identified, such as age, gender, education level, social grade/occupation, marital status, training, type of CPR training, length of time since last training, number of times trained, belief that the public should learn how to use AEDs, confidence in performing CPR, and legal obligation under emergency medical service law. Collectively, these can be summarized into socio-demographics, training, attitudes, perceived norms, self-efficacy, and legal obligation factors (Table 4).

**Table 4:** Factors analysed in each of the included studies.


FACTORS		AUTHOR	FACTORS INFLUENCING WILLINGNESS TO PERFORM CPR/USE AN AED	STRENGTH OF ASSOCIATION/SIGNIFICANCE LEVEL

**Socio-demographics**				

Age	Younger age	Hawkes et al. [24]	aged 18 to 34 years are less likely to phone EMS compared with those aged ≥35 years	– OR: 0.46 (95% CI: 0.30–0.69)

			aged 18 to 34 years were significant indicators had undergone any type of training and training in CPR, both in the past 5 years.	– OR 1.63 (95% CI: 1.27–2.08)

		Krammel et al. [25]	age <45 years are highest willingness to perform CPR compared to 45 years and above	– (<45: 40% vs. >45: 31%; OR: 0.72 [95% CI: 0.57 ± 0.92]; p = 0.027)

			age <45 years are highest willingness to use AED compared to 45 years and above	– (<45: 57% vs. >45: 54%; OR: 0.68 [95% CI: 0.54 ± 0.85]; p = 0.001)

		Qian et al. [31]	18–29 years	– 2.53 ± 0.52 (p < 0.001)

		Son et al. [33]	the rates of willingness to perform CPR decreased with older age	– 20 to 29 years (64.6%), 50 to 59 years (59.8%), 60 years or above (38.6%)

Gender	Male	Son et al. [33]	male respondents reported a willingness to perform CPR	– 67.5% among male respondents

		Lee et al. [26]	willingness to use AEDs were male	– AOR, 1.39 (95% CI: 1.10–1.75

		Pei-Chuan Huang et al. [28]	men were more likely to perform CPR than women	– OR: 2.34, p = 0.005

		Pei-Chuan Huang et al. [29]	men were more likely use AED	– OR: 2.37, p = 0.007

		Hawkes et al. [24]	women were less likely than men to go and get an AED, and use an AED	– go and get an AED (OR: 0.80) (95% CI: 0.66–0.97) – use an AED (OR: 0.63) (95% CI: 0.51–0.78)

		Krammel et al. [25]	female individuals reported a significantly lower willingness to initiate CPR and to use an AED device	– initiate CPR (male: 40% vs. female: 25%; OR: 2.03 [95% CI: 1.39 ± 2.98]; p < 0.001) – use an AED device (male: 58% vs. female: 44%; OR: 1.76 [95% CI: 1.26 ± 2.53]; p = 0.002)

	Women	Cheskes et al. [22]	women were more likely indicate a willingness to perform CPR	– OR: 2.3, 95% CI [1.4, 3.8]

Education Level	Higher education level	Son et al. [33]	college graduates or respondents with a higher level of education.	– 68.9% of 560 college graduates or respondents with a higher level of education (p < 0.001)

		Qian et al. [31]	postgraduate and above	– willingness to rescue 2.50±0.53 (p < 0.001) – willingness to rescue under professional guidance 2.83 ± 0.40 (p < 0.001) – willingness to rescue after learning BLS 2.73 ± 0.47 (p < 0.001)

Social grade/Occupation		Shams et al. [32]	earning higher income	– p = 0.009

		Hawkes et al. [24]	social grade A, B, or C1*	– OR: 1.70 (95% CI: 1.17–2.47)

		Qian et al. [31]	different type of occupation	– p < 0.0001

Marital status	Married	Hawkes et al. [24]	married or living as married is a predictive factor to perform CO-CPR, CPR and were more likely to go and get an AED	– perform CO-CPR (OR: 1.30) (95% CI: 1.07–1.57) – perform CPR (OR: 1.35) (95% CI: 1.10–1.66 – go and get an AED (OR: 1.4) (95% CI: 1.10–1.80

**Training**				

Type of CPR training	Interest to participate in CPR training course and belief that the public should participate in CPR training courses	Pei-Chuan Huang et al. [28]	those who expressed interest in attending a course	– OR: 2.79, p = 0.001

			those who believed that the public should participate in CPR training courses	– OR: 2.84, p = 0.048

	Interest to participate in an AED training course and belief that the public should learn how to use AEDs	Pei-Chuan Huang et al. [29]	those who expressed interest to participate in a training course	– OR: 3.14, p = 0.001

			those who believed that the public should learn how to use AEDs	– OR: 5.06, p < 0.001

	Previous CPR training	Shams et al. [32]	previous CPR training were significant predictors of willingness to perform CPR in the event of a cardiac arrest	– OR: 1.627 (95% CI: 1.018–2.600) (p = 0.042)

	First aid experience and without first aid experience	Qian et al. [31]	those with first aid experience were more willing to attempt rescue than those without first aid experience	– 66.72% of 445 respondent with first aid experience were more willing to attempt rescue (p < 0.001)

	First aiders with and without AED training	Fan et al. [23]	those with AED training were also more likely to commence CPR and were more likely to try to locate an AED and apply it	– perform CPR (47.4% vs 8.0%, p < 0.001) – try to locate an AED (53.3% vs 17.4%, p < 0.001) – apply AED (41.6% vs 5.7%, p < 0.001)

	Instructor-led training and self-learning training	Bylow et al. [21]	instructor-led training resulted in a statistically significant higher total score, self-assessed knowledge, and willingness to act immediately compared to self-learning training	– median 61 vs 59, p < 0.0001

	Didactic plus practise group and didactic only group	Son et al. [33]	the rate of CPR willingness in the didactic plus practice group was significantly higher than that in the didactic only group	– 79.9% vs 63.9%

			didactic plus practise group found to be significantly associated with CPR willingness compared to didactic only group	– AOR: 3.38 (95% CI: 2.3-5.0)

	CCO-CPR and traditional CPR with MTM ventilation	Cheskes et al. [22]	the proportion of respondents willing to provide CCO-CPR was significantly greater than the proportion of respondents willing to perform traditional CPR with MTM ventilations	– the unknown OHCA victim (61.5% vs 39.7%, p < 0.001), stranger (55.1% vs 38.8%, p < 0.001) and unkempt/homeless individual (47.9% vs 28.5%, p < 0.001)

	High school coaches and club coaches	Post et al. [30]	high school coaches having greater levels of emergency preparedness for immediate medical care during practices and competitions than club sport coaches	– 58.6% high school coaches had trained in three categories CPR training, AED training or first-aid training compared with 23.9% of club coaches (X2 = 84.9, P < .001).

	CCO-CPR, CPR and PAD training	Hawkes et al. [24]	having ever trained in CPR (CO-CPR and/or CPR) was the most important factor in the willingness of an individual to call EMS	– OR 9.18 (95% CI: 4.39–19.23)

			having ever trained in CPR (CO-CPR and/or CPR) was the most important factor in the willingness of an individual to perform CPR	– OR: 5.39 (95% CI: 4.29–6.76)

			training in defibrillator use ever were the most significant predictive factors for an individual’s willingness to go and get or use an AED	– OR: 2.62 (95% CI: 1.71–4.01)

Length of time since last training		Lee et al. [26]	CPR training experience in the previous 2 years were associated with willingness to use AEDs	– AOR: 1.80 (95% CI: 1.43–2.28)

		Son et al. [33]	Interval of less than 6 months	– AOR was 4.47 (95% CI: 1.29-15.52) for intervals shorter than 6 months

			AED training was included in CPR education	– AOR for CPR willingness was 5.98 (95% CI: 2.30-15.53)

		Hawkes et al. [24]	training in CPR in the past 5 years	– OR: 1.37 (95% CI: 1.04–1.80)

			training in AED in the past 5 years	– OR: 2.26 (95% CI: 1.32–3.89) – use an AED 5.20 (95% CI: 3.07–8.82)

Number of times trained		Son et al. [33]	group receiving CPR education on 4 or more occasions	– AOR: 7.68 (95% CI: 3.21-18.35)

**Attitude**	Positive attitude	Pei-Chuan Huang et al. [29]	“belief that the public should learn how to use AEDs” were the independent factors associated with a higher willingness among bystanders to use AEDs	– OR: 5.06, p < 0.001

**Perceived norms**	Social pressure	Lee et al. [26]	perception of “awareness of how to use an AED”	– AOR: 4.40 (95% CI: 3.26–5.93)

		Liaw et al. [27]	perception of “using AED is important for unresponsive victims”	– z = 4.32, p < 0.001

			perception of “AED practice drills should be performed on a regular basis”	– z = – 2.41, p = 0.02

**Self-efficacy**	Perceptions of their capacity to engage in the behaviour with a predictable result	Shams et al. [32]	confident in one’s ability to apply an AED, or perform CPR.	– perform CPR (OR: 1.93) (95% CI: 1.285–2.898) (p = 0.002) – apply an AED (OR: 1.761) (95% CI: 1.021–3.036) (p = 0.042)

		Son et al. [33]	confidence in performing CPR	– 95.3% of 85 respondents (p < 0.001)

		Hawkes et al. [24]	having witnessed an arrest was associated with a greater likelihood to perform CPR and to use an AED	– to perform CPR (OR: 1.53) (95% CI: 1.17–2.01) – to use an AED (OR: 1.61) (95% CI: 1.23–2.12)

		Liaw et al. [27]	increase the confidence to perform CPR, use AED, identify victims with no signs of life, and the willingness to perform CPR and AED without hesitancy	– increase the confidence to perform CPR (z = – 8.56, p< 0.001) – use AED (z = – 8.93, p < 0.001) – identify victims with no signs of life (z = – 7.88, p < 0.001) – and the willingness to perform CPR and AED without hesitancy (z = – 8.91, p < 0.001)

**Legal obligation**		Son et al. [33]	increased the respondent’s willingness to perform CPR in South Korea under Korean EMS laws	– 66.9% of 130 respondents reported their willingness, (p = 0.017)

		Lee et al. [26]	increased the respondent’s willingness to use AEDs in South Korea under Korean EMS laws	– AOR: 1.45 (95% CI: 1.13–1.86)

		Pei-Chuan Huang et al. [28]	increased the respondent’s willingness to perform CPR in Taiwan under limited Good Samaritan immunity	– 85.4% of 916 respondent is reported their willingness


Notes: *High managerial, administrative, or professional; B: intermediate managerial, administrative, or professional; C1: supervisory, clerical, and junior managerial, administrative, or professional.

The common factors reported that were more likely to indicate a willingness to perform CPR and use an AED are younger age below 45 years old, men, higher level of education, employed, being married/living as married, having trained in CPR and AED in the previous 5 years, having received CPR education on four or more occasions, having a positive attitude and perception toward CPR and AED, having confidence to perform CPR and to apply an AED, and legal liability protection under EMS law.

### 3.3 Community barriers

There are seven studies identified barriers in the included studies that can impact respondents’ willingness to perform CPR and use an AED [22,26,27,28,29,31,32].

The reluctance of performing CPR and AED varies between studies and different countries. Barriers that are often found in the included studies were fear of litigation or legal responsibilities [22,26,28,29,31], injuring victim due to performing CPR [27,28,31,32], and injuring victim due to using AED [26,27,29] (Table 5).

**Table 5:** Barriers analysed in each of the included studies.


BARRIERS	STUDIES

Lack of knowledge	Cheskes et al. [22]; Shams et al. [32]; Lee et al. [26]

Fear of litigation/legal responsibilities	Cheskes et al. [22]; Pei-Chuan Huang et al. [28]; Lee et al. [26]; Pei-Chuan Huang et al. [29]; Qian et al. [34]

Lack of skill confidence	Cheskes et al. [22]; Pei-Chuan Huang et al. [29]

Fear of disease transmission	Shams et al. [32]; Liaw et al. [35]; Qian et al. [34]

Injuring victim due to performing CPR	Shams et al. [32]; Pei-Chuan Huang et al. [28]; Liaw et al. [35]; Qian et al. [34]

Injuring own self due to performing CPR	Liaw et al. [35]

Injuring victim due to using AED	Liaw et al. [35]; Lee et al. [26]; Pei-Chuan Huang et al. [29]

Not interested in AED	Lee et al. [26]

Injuring own self due to using AED	Liaw et al. [35]

Poor hygiene of victim	Shams et al. [32]

Presence of vomit or blood	Shams et al. [32]

Performing mouth-to-mouth artificial respiration	Qian et al. [34]

Concern that peoples around them will complain	Qian et al. [34]

Unwillingness to touch strangers	Qian et al. [34]


## 4. Discussion

The present systematic literature review analysis has identified six factors from the included studies—socio-demographics, training, attitudes, perceived norms, self-efficacy, and legal obligation—that are associated with the willingness to perform CPR and use AED.

The community’s willingness to rescue is higher among younger populations. Many educational initiatives, particularly those geared toward the younger generation (such as the first aid course), have influenced the level of awareness and knowledge in this age group. The availability of information, the acceptance of new knowledge, and physical features could also be contributing factors. It is well-known that elderly people are substantially less willing to perform CPR and use an AED. To persuade a greater proportion of the elderly to perform CPR and use an AED on a cardiac arrest victim, public health interventions must target their attitudinal beliefs, which include the following characteristics: the individuals who are most likely to adopt these behaviors are already aware that CPR can save a life; that mastering this skill can be rewarding; that CPR should be initiated immediately, even before EMS arrives; and that they are unlikely to be punished for attempting to assist someone [36]. The first aid module designed specifically for the elderly can educate and fill the gap of knowledge among them, and persuade elderly individuals about the importance of CPR and AED, which are most likely to have an impact on this community [25].

Interestingly, most of the studies found that men are significantly more willing to perform CPR and use AEDs than women, according to the gender-related findings, which shows that there is a significant gender gap in this area. We suggest that encouraging, empowering, and educating more women to administer CPR and use an AED device in emergency situations could help lessen some of this disparity. Furthermore, we discovered a higher education level and a higher social grade with a willingness to perform CPR and AED use. Higher education level may be related to the higher acceptance of new knowledge, greater access to information, and physical factors [31]. Besides, higher social grade can be related to their awareness of CPR as well as the fact that they are in a position of responsibility for other employees [35]. Consideration should be given to implementing targeted training programs to reach more individuals working in unskilled jobs and residing in impoverished areas [24].

In contrast, the current strategy for increasing CPR and AED use places emphasis on training. Combined CPR and AED training seems to be better than training in CPR alone and is a significant positive factor associated with the willingness to act in the event of witnessing an OHCA. To increase the community’s willingness to perform CPR and AED use, analyses of the factors related to CPR and AED training would become crucial. We propose that priority groups for training include women, the unemployed, those of lower social status, and the elderly. Awareness of the importance of CPR training, potential health problems in relatives and friends, and free training were the top three motivators for CPR training [36].

The legal obligation imposed by emergency medical service law was found to have a significant relationship with community willingness to perform CPR and use an AED. The laws regarding emergency care will promote bystander CPR rates by protecting laypeople or bystander rescuers from any civil or criminal liability if the patient dies [37]. In line with the effectiveness of the introduction of this law, three studies in South Korea [26,33,37] and one study in Taiwan [28] have found an association between the recognition of the Good Samaritan law and the willingness to perform CPR and to use AEDs on a stranger.

One of the main barriers that influence the community’s willingness to attempt rescue is fear of litigation, which refers to the absence or imperfection of relevant legislation. This disparity could be attributable to differences in legislation (such as Good Samaritan laws) and the litigious culture of other nations [22]. This suggests that the government implements legal liability protection to reduce concerns about legal liability among those who are willing to help, and education about the emergency medical service legislation to promote bystander CPR and the use of public AEDs.

Another frequently reported barrier is worry about injuring the victim due to performing CPR and using AED. We need to stress that CPR and the use of an AED are unlikely to result in major injuries, that delays in CPR and the use of an AED can result in permanent brain damage to victims of sudden cardiac arrest, that the chances of the victim’s survival can be increased by two to four times by performing CPR, and that using an AED can increase survival by 50–70% [11,28].

Interventions can then be designed to address the specific determinants of the intention to perform CPR and use an AED. Training in first aid and AED seems to be better than training in first aid alone. In a study conducted in Hong Kong by Fan et al. [23], first aiders were found to have a more positive attitude toward responding to a cardiac arrest victim and to be more knowledgeable about the use of an AED compared with non–first aiders. Also, first aiders with AED training were more likely to respond to an OHCA victim, provide life-support intervention, be more knowledgeable about AEDs, and try to locate an AED and apply, compared with first aiders without AED training [23]. This mode of intervention suggests the importance of community-based education and training about sudden cardiac arrest and basic life support, including the use of an AED.

We encourage the public to initiate chest-compression-only CPR (CCO-CPR), also known as ‘hands-only’ CPR, first when confronting OHCA. CPR guidelines recommend CCO-CPR for both untrained and trained bystanders unwilling to perform rescue breathing. CCO-CPR was introduced by the American Heart Association in 2010 to allow non-medical professionals to perform CPR and to encourage a larger population to participate in first aid [38]. Non-medical professionals can use simple compression CCO-CPR because it is easier to teach and perform than traditional CPR with MTM ventilations, which is more intimate and makes one’s reluctance stronger [5]. Highlighting the simplification of CPR through promoting the ‘hands-only’ CPR technique may encourage more people to render assistance in an emergency and to undertake training. CCO-CPR has streamlined the learning process and made it easier for the general public to overcome many of the stated barriers, such as MTM resuscitation, CPR technique complexity, lack of confidence, hygiene, and fear of disease transmission [39]. The concept of CCO-CPR has emerged as an alternative to traditional CPR with MTM ventilation, and CCO-CPR is an option in future CPR guidelines because it is associated with increased CPR rates and a higher rate of overall cardiac arrest survival [40].

There are several initiatives can be taken to facilitate bystander CPR and AED use, such as widespread hand-only CPR in large-scale training or awareness events (e.g., mass CPR events, CPR and AED education at marathon races), establishing first-responder programs in local communities, supply of AED at public places that have a high density of citizens (e.g., shopping malls, sport facilities, airport, railway stations, bus terminals), crowd sourcing to identify AED locations (e.g., use of mobile information technology such as an AED locator application), linking AEDs to emergency dispatch centers, efforts of improving dispatcher-assisted interventions to overcome such difficulties (e.g., dispatcher-prompted CPR, dispatcher-activated alert of nearby volunteers via text message), more frequent EMS dispatch, and shortening the training course in one hour to increase the public willingness to attend [15,23,28,29,41,42].

Policymakers should concentrate their future efforts on enhancing public information campaigns, public CPR and AED education, awareness of cardiac arrests, encouraging the appropriate and prompt initiation of CPR in patients experiencing OHCA, facilitating the distribution and access to AEDs, and education about the Good Samaritan law [43,44]. Educational interventions and first aid training courses should be highlighting that CCO-CPR has recently been made much simpler and is acceptable for bystander CPR, that CPR and use of an AED are not likely to result in major injuries, that the most likely victim could be a family member, that delays in CPR and AED can result in permanent brain damage to victims of sudden cardiac arrest, and that the chances of the victim’s survival can be increased by two to four times with minimal risk to the rescuer [11,28]. Also, the government should take appropriate policy development into consideration in order to increase CPR competency in the community. One suggestion is mandatory community-level training strategies, such as adding CPR and AED training to the educational system in school, college, and driver’s license requirements to ensure that a large proportion of the community receives CPR and AED training at least once in their lifetime to improve the ability of bystanders to perform CPR and use an AED [28,45,46].

## 5. Strengths and limitations

To the best of our knowledge, this is the first comprehensive analysis of factors and barriers that influence community willingness to perform CPR and use an AED. This review compiles a large amount of information on the factors that influence the community to perform CPR and use an AED, as well as the possible reasons for the low rates of bystander-initiated CPR and AED in global populations. It provides an idea of what the contributing factors and main barriers are to successful CPR and AED implementation. This will allow all stakeholders to consider the interventions to overcome these barriers, thus contributing to the success of intervention programs.

This systematic review has several limitations. First, the majority of the included studies focused on developed and developing countries. There are limited studies related to CPR and AED willingness among less developed countries. Further analysis of the relationship between factors and barriers influencing community willingness to perform CPR and use an AED requires additional data from other countries. Second, the findings of this review were restricted to a particular search engine chosen by the authors based on the frequency of articles relevant to the research topic. It cannot be ruled out that other studies that were not included in the chosen database were overlooked. Third, only English-language publications were included. As a result, publications written in languages other than English were ignored, which may have contributed to selection bias. Finally, systematic review articles, conference proceedings, book chapters, and reports were excluded because we were unable to adequately assess the quality of such research.

## 6. Conclusion

This review revealed the important role of the community in performing CPR and using AED before EMS arrival to improve OHCA victims’ survival. A total of 13 studies were reviewed, and it can be concluded that there are six factors influencing community willingness: socio-demographics, training, attitude, perception, behavior, and legal obligation. The role played by all stakeholders should be strengthened to ensure the success of intervention programs. Among the urgent measures that need to be taken are ongoing and frequent CPR and AED education intervention programs, as well as the formulation of policies and laws to require the community’s implementation of CPR and AEDs. Measures that should be considered include the ongoing efforts based on the successful implementation of programs in other countries that have successfully increased the implementation of CPR and AEDs among the community and successfully increased OHCA survival. This review may provide guidance to the stakeholders to empower the community to perform CPR and use AED, and thus indirectly reduce morbidity and mortality rates and increase OHCA survival.

## Additional Files

The additional files for this article can be found as follows:

10.5334/gh.1255.s1 Appendix 1. Full search string used in selected databases.

10.5334/gh.1255.s2 Appendix 2. The criteria used to determine the rigor of the methodology and analysis used in the selected articles.
