# Supplementary material for: Factors and Barriers on Cardiopulmonary Resuscitation and Automated External Defibrillator Willingness to Use among the Community: A 2016–2021 Systematic Review and Data Synthesis
Source: Glob Heart. 2023 Aug 25;18(1):46. doi: 10.5334/gh.1255 (PMC10464530; doi:10.5334/gh.1255)
Supplement: Appendix 1. — Full search string used in selected databases. [file gh-18-1-1255-s1.pdf]

Appendix 1: Full search string used in selected databases.

Database	Search string
Scopus	TITLE-ABS-KEY(("cardiopulmonary resuscitation" OR "CPR" OR "basic life support" OR "BLS" OR "basic cardiac life support" OR "Mouth-to-Mouth Resuscitation" OR "Code Blue") AND ("automated external defibrillator" OR "AED" OR "public access defibrillator" OR "PAD" OR "defibrillator*" OR "Electric Shock Cardiac Stimulator*") AND ("read*" OR "willing*" OR "prepare*") AND ("community*" OR "public*" OR "people*" OR "bystander*" OR "society" OR "population*") AND ("barrier*" OR "reluctant" OR "boundary" OR "factor*" OR "attitude*" OR "behaviour"))
WOS	TS=(("cardiopulmonary resuscitation" OR "CPR" OR "basic life support" OR "BLS" OR "basic cardiac life support" OR "Mouth-to-Mouth Resuscitation" OR "Code Blue") AND ("automated external defibrillator" OR "AED" OR "public access defibrillator" OR "PAD" OR "defibrillator*" OR "Electric Shock Cardiac Stimulator*") AND ("read*" OR "willing*" OR "prepare*") AND ("community*" OR "public*" OR "people*" OR "bystander*" OR "society" OR "population*") AND ("barrier*" OR "reluctant" OR "boundary" OR "factor*" OR "attitude*" OR "behaviour"))
Medline Complete via Ebscohost	("cardiopulmonary resuscitation" OR "CPR" OR "basic life support" OR "BLS" OR "basic cardiac life support" OR "Mouth-to-Mouth Resuscitation" OR "Code Blue") AND ("automated external defibrillator" OR "AED" OR "public access defibrillator" OR "PAD" OR "defibrillator*" OR "Electric Shock Cardiac Stimulator*") AND ("read*" OR "willing*" OR "prepare*") AND ("community*" OR "public*" OR "people*" OR "bystander*" OR "society" OR "population*") AND ("barrier*" OR "reluctant" OR "boundary" OR "factor*" OR "attitude*" OR "behaviour")

Notes:
The symbol * was used in the search strategy as truncation and wildcard function to increase variability of selected keywords.
