# Supplementary material for: Factors and Barriers on Cardiopulmonary Resuscitation and Automated External Defibrillator Willingness to Use among the Community: A 2016–2021 Systematic Review and Data Synthesis
Source: Glob Heart. 2023 Aug 25;18(1):46. doi: 10.5334/gh.1255 (PMC10464530; doi:10.5334/gh.1255)
Supplement: Appendix 2. — The criteria used to determine the rigor of the methodology and analysis used in the selected articles. [file gh-18-1-1255-s2.pdf]

Appendix 2. The criteria used to determine the rigor of the methodology and analysis used in the selected articles.

**Research design**	**Assessment criteria**
Qualitative	QA1-Is the qualitative approach appropriate to answer the research question?<br>QA2-Are the qualitative data collection methods adequate to address the research question?<br>QA3-Are the findings adequately derived from the data?<br>QA4-Is the interpretation of results sufficiently substantiated by data?<br>QA5-Is there coherence between qualitative data sources, collection, analysis and interpretation?
Quantitative randomized controlled trials	QA1-Is randomization appropriately performed?<br>QA2-Are the groups comparable at baseline?<br>QA3-Are there complete outcome data?<br>QA4-Are outcome assessors blinded to the intervention provided?<br>QA5-Did the participants adhere to the assigned intervention?
Quantitative (non-randomised)	QA1-Are the participants representative of the target population?<br>QA2-Are measurements appropriate regarding both the outcome and intervention (or exposure)?<br>QA3-Are there complete outcome data?<br>QA4-Are the confounders accounted for in the design and analysis?<br>QA5-During the study period, is the intervention administered (or exposure occurred) as intended?
Quantitative (descriptive)	QA1-Is the sampling strategy relevant to address the research question?<br>QA2-Is the sample representative of the target population?<br>QA3-Are the measurements appropriate?<br>QA4-Is the risk of nonresponse bias low?<br>QA5-Is the statistical analysis appropriate to answer the research question?
Mixed methods	QA1-Is there an adequate rationale for using a mixed methods design to address the research question?<br>QA2-Are the different components of the study effectively integrated to answer the research question?<br>QA3-Are the outputs of the integration of qualitative and quantitative components adequately interpreted?<br>QA4-Are divergences and inconsistencies between quantitative and qualitative results adequately addressed?<br>QA5-Do the different components of the study adhere to the quality criteria of each tradition of the methods involved?
